# Survival and risk factors of mortality among patients with TB/HIV coinfection in Kigali City, Rwanda: a retrospective cohort study (2019–2024)

**DOI:** 10.1186/s12879-026-13744-2

**Published:** 2026-06-05

**Authors:** Didier Ndabana, Solange Nikwigize, Felix Murego, Jerome Ndayisenga, Patrick Migambi, Amanuel Kidane Andegiorgish

**Affiliations:** 1 Tuberculosis and HIV, Nyamata Level 2 Teaching Hospital, Nyamata, Bugesera +250 Rwanda; 2 Department of Epidemiology and Biostatistics, School of Public Health, College of Medicine and Health Sciences, University of Rwanda, Remera, Kigali +250 Rwanda; 3 Tuberculosis and Other Respiratory Diseases (TB&ORD) Division, Rwanda Biomedical Centre, Kimihurura, Kigali Rwanda; 4 National Reference Laboratory, Rwanda Biomedical Centre, Rwanda Biomedical Centre, Nyarugenge, Kigali +250 Rwanda

**Keywords:** Tuberculosis, TB/HIV coinfection, Mortality, Survival analysis, Rwanda

## Abstract

**Background:**

Tuberculosis (TB) is still the most common cause of mortality among people living with HIV (PLHIV) worldwide. However, in Rwanda, no study has specifically assessed the survival and risk factors for mortality among patients with TB/HIV co-infection in urban settings such as Kigali city. This study aims to determine the survival probability and identify risk factors for mortality among patients with TB/HIV coinfection in Kigali City, Rwanda.

**Methods:**

A retrospective cohort study was conducted among 1,966 patients aged (≥ 15 years) with TB/HIV co-infection who were receiving antiretroviral therapy (ART) at the time of TB diagnosis and were enrolled at the initiation of treatment for drug-susceptible tuberculosis at 48 health facilities in Kigali between July 2019 and March 2024. Data were extracted from the e-TB system and patients’ medical records. The survival probability up to 168 days was estimated via the Kaplan–Meier method. Cox proportional hazards regression was used to predict mortality. Statistical significance was set at *p* < 0.05.

**Results:**

The median age of the patients was 39 years (IQR: 33–46), and 66.0% were male. A total of 264 patients (13.4%, 95% CI: 11.9–15.0) died, and 78.8% of the deaths occurred within the first 56 days of follow-up. The overall survival probability was 86.6% (95% CI: 84.9–88.0). Significant predictors of mortality included age ≥ 45 years (adjusted hazard ratio [aHR] = 2.21; 95% CI: 1.18–4.11), no receipt of cotrimoxazole preventive therapy (CPT) (aHR: 1.65; 95% CI: 1.29–2.13), the presence of comorbidities(including diabetes mellitus, hypertension, asthma, chronic liver disease, chronic kidney disease, malignancy, and other chronic illnesses) (aHR: 3.18; 95% CI: 2.27–4.45), no receipt of tuberculosis preventive treatment (TPT) (aHR: 1.87; 95% CI: 1.38–2.54), undernutrition (aHR: 2.72; 95% CI: 2.12–3.50), fair ART adherence (aHR: 1.89; 95% CI: 1.25–2.87), low CD4 count < 200 cells/mm³ (aHR: 2.30; 95% CI: 1.74–3.05) and extrapulmonary TB (aHR: 1.62; 95% CI: 1.15–2.28).

**Conclusion:**

Mortality among patients with TB/HIV-coinfected in Kigali remains high, particularly during the early treatment period. Predictors of mortality included older age, lack of TPT and CPT, comorbidities, undernutrition, fair ART adherence, low CD4 count and extrapulmonary TB. Increasing TB/HIV integrated care through improved adherence support, nutritional supplementation, comorbidity management, increased uptake of cotrimoxazole and TPT, and timely interventions are critical to reduce mortality.

**Clinical trial number:**

Not applicable.

## Background

 Tuberculosis (TB) and human immunodeficiency virus (HIV) are still two of the most important public health problems worldwide and carry a heavy burden [1]. The dual burden of these diseases exacerbates the disease burden, leading to increased morbidity and mortality in affected populations [2]. TB is currently the leading cause of death among people living with HIV and was responsible for approximately 30% of all AIDS-related deaths worldwide in 2019, accounting for approximately 208,000 fatalities [3]. The highest burden occurs in the African and Southeast Asian regions, with 82% of the combined total TB deaths in people without HIV and people living with HIV (PLWH) [2]. In 2020, the estimated annual TB incidence in the sub-Saharan Africa region was 220 per 100,000 people, although PLWH were found to have a significantly higher incidence rate, approximately 2,279 per 100,000 per year [1]. A systematic review and meta-analysis revealed that the overall HIV prevalence among TB patients in the sub-Saharan region was 31.8%, with variations by subregion: southern Africa, 43.7%; central Africa, 41.3%; eastern Africa, 31.1%; and western Africa, 25.5% in 2019 [4].

The interaction between TB and HIV is bidirectional, as one disease can directly affect the treatment and outcomes of the other disease [4, 5]. People living with HIV are at a much greater risk for developing active TB, with estimates 15–21 times greater than those of people without HIV [6]. The reason for this increased susceptibility is attributed to the effects of HIV, which suppresses the immune response to Mycobacterium tuberculosis [6, 7].

The implementation of TB/HIV management strategies, such as TB and HIV service integration and antiretroviral therapy (ART), has progressed, yet mortality among patients with TB/HIV-coinfected remains unacceptably high in low-resource settings [8]. Like many other high-burden countries, Rwanda has made good progress in scaling up antiretroviral therapy (ART) and integrating TB/HIV services; the country has achieved high ART coverage and has implemented routine HIV testing for all TB patients and TB screening for all PLHIV [9, 10]. However, TB/HIV coinfection continues to cause considerable mortality, particularly in Kigali city [11].

Previous studies in various settings have established clinical and demographic predictors of poor outcomes among patients with TB/HIV coinfection. Immunodeficiency (CD4 < 200 cells/mm³), WHO stage IV disease, being bedridden, the absence of prophylactic drugs, old age and substance abuse are predictors of poor survival [7, 12–17]. In addition, prior studies have shown that mortality is highest in the early months of anti-TB treatment, and survival analysis studies underscore the role of timely and effective interventions for patients in the early months of treatment [13, 14].

In Rwanda, no study has assessed the survival of patients with TB/HIV-coinfected, especially in urban settings such as Kigali city, which may have different patient characteristics than rural settings do. Understanding survival and identifying risk factors associated with mortality in patients with TB/HIV-coinfected is important for improving patient management and enhancing the integration of TB/HIV collaborative care. Therefore, this study aims to determine the survival probability and identify risk factors for mortality among patients with TB/HIV coinfection in Kigali City, Rwanda. The findings are expected to help inform clinical decision-making and advance evidence-based policy development within Rwanda’s national TB and HIV programs.

## Methodology

### Study design, setting and period

A retrospective cohort study involving patients with TB/HIV coinfection was carried out from July 2019 to March 2024 in Kigali city, Rwanda. Participants entered the cohort at the time of the initiation of TB treatment while already receiving antiretroviral therapy (ART). Follow-up started on the date of TB treatment initiation and continued until death, loss to follow-up, transfer-out, or treatment completion.

Kigali, the capital city of Rwanda, has an estimated population of 1,745,555 million, as reported in the 2022 Census [18], and comprises three districts (Nyarugenge, Gasabo, and Kicukiro). The city has 48 health facilities providing services for the treatment of TB, which include 7 hospitals (4 district hospitals: Muhima, Nyarugenge, Kibagabaga, and Masaka; and 3 referral hospitals: CHUK, King Faisal Hospital, and Rwanda Military Hospital) and 41 health centers distributed across the three districts of Kigali (Gasabo, Kicukiro, and Nyarugenge). Each of these health facilities offers ART and has the capacity to diagnose, treat, and monitor TB patients.

### Study population and sampling

Patients were screened for inclusion based on the following criteria:

#### Inclusion criteria

(1) Patients aged (≥ 15 years) living with HIV; (2) with documented TB/HIV coinfection; (3) already receiving ART at the time of TB diagnosis; (4) diagnosed with drug-susceptible TB; and (5) initiation of TB treatment between 1 July 2019 and 31 March 2024.

#### Exclusion criteria

(1) Patients living with HIV aged < 15 years; (2) patients not receiving ART at the time of TB diagnosis; (3) patients with multidrug-resistant TB (MDR-TB); (4) patients treated with drug-susceptible TB for more than 168 days (e.g., TB meningitis, osteoarticular TB); and (5) Patients with missing information on key variables, including TB diagnosis date, TB treatment initiation, and TB treatment outcomes.

TB/HIV co-infection was ascertained using a two-step process: (i) TB diagnosis was confirmed by either bacteriological confirmation (Xpert MTB Detected / RIF Not Detected, positive smear microscopy, or culture) or clinical diagnosis according to national TB guidelines [19]; (ii) HIV-positive status was confirmed by documented evidence in the e-TB system and validated through a review of ART registers, TB registers and TB treatment cards. Only patients with both confirmed TB and documented HIV-positive status were included in the cohort.

An a priori sample size calculation was not performed; instead, an exhaustive enumeration approach was used, whereby all available records of patients with TB/HIV co-infection during the study period were included in the analysis.

### Data collection tools and procedure

This study used secondary data from all 48 health facilities that provide tuberculosis (TB) treatment and antiretroviral therapy (ART) services in Kigali city. Patients with TB/HIV co-infection were identified by cross-referencing the electronic TB recording system (e-TB) with HIV/AIDS services records; specifically, all patients diagnosed with drug-susceptible TB were included only if they also had documented HIV-positive status confirmed in either the ART registry, electronic medical records (EMRs), or a documented HIV test result in their TB register and TB treatment card. The data were obtained from the electronic TB recording system (e-TB) within the Health Management Information System (HMIS). The main host institution for the centralized electronic records was the Rwanda Biomedical Centre (RBC), specifically through its Tuberculosis and Other Respiratory Diseases (TB&ORD) Division, which manages the national e-TB system.

This information was supplemented with information from TB registries, ART records, TB treatment cards, hospitalization records, laboratory results, electronic medical records (EMRs), and death audit reports hosted at and accessed from the 48 individual health facilities in Kigali city.

Mortality outcomes were ascertained from the e-TB/HMIS database and cross-validated onsite using multiple facility-level data sources. These included patient medical files, paper-based TB registries, and institutional death audit reports.

All the data were anonymized, and no personal identifiers were collected. Each HMIS record had a unique system-generated identifier to link patient information across non-HMIS data sources.

Data from non-HMIS sources were collected via a Kobo-based electronic data extraction tool that aimed to capture all relevant study variables and complement the information from the HMIS e-TB records. Ten trained healthcare professionals extracted the data over a period of time from 19 April–6 June 2025. The healthcare professionals who extracted the data received standardized training from the (TB&ORD) Division in partnership with the University of Rwanda. This training included the use of extraction tools, ethical considerations, and study procedures. The Nurse-in-Charge of TB/HIV services supported the process on site, while two staff members from the National Tuberculosis Program supervised it. The data extraction tools were adapted from literature and customized to gather information from patient charts, EMRs, ART files, and TB treatment cards.

### Study variables

#### Dependent variable

Mortality status (whether the patient survived or died during the study period).

Time-to-event (time from TB/HIV coinfection diagnosis or treatment initiation to the occurrence of death or the end of the study period, measured in days).

#### Independent variables

##### Sociodemographic factors

Age, sex, marital status (married/cohort, single, widowed, divorced), educational status (tertiary, secondary, primary, illiterate), and occupation (formal employment, informal sector, unemployed).

Clinical characteristics: CD4 count/mm, nutritional status (BMI), site of disease, comorbidities (DM, HTN, asthma and other), TB method of confirmation, and site of disease.

##### Treatment-related factors

Adherence to ART, Cotrimoxazole (CPT), Tuberculosis preventive treatment (TPT) uptake, Among the variables listed above, TPT uptake, occupation, education status, marital status, and TB/HIV treatment adherence data (including corrected outcome dates) were retrospectively collected onsite from HIV and TB patient tools. These data complemented existing electronic records extracted from the E_TB/HMIS system since they are not included in that system.

### Operational definition

#### Survival time

The time from the entry of each individual into the cohort (TB treatment initiation) up to the event of interest  (death) or censoring.

#### Follow-up period

The period of time during which participants in a study are followed from the date of TB treatment up to the endpoint of 168 days to monitor outcomes of interest, or survival. The 168-day (approximately 6 months) follow-up period was selected based on the standard duration of treatment for drug-susceptible TB according to national TB treatment guidelines in Rwanda [19]. This ensured a uniform observation period across participants and alignment with programmatic treatment outcomes.

#### Events

The event of interest is the occurrence of the outcome of interest during the follow-up period; in this study, the event is the death of patients with TB/HIV-coinfected.

#### Censored

A patient who did not develop an event (death) and was still alive at the end of the follow-up period.

#### Loss to follow-up

A patient with TB/HIV-coinfected whose treatment was interrupted for ≥ 2 consecutive months.

#### Survival rate

percentage of patients alive after a specified follow-up time period. This allows us to measure the effects of different factors on patient outcomes.

#### Drug-susceptible TB

is a tuberculosis infection caused by Mycobacterium tuberculosis bacteria that can be killed by standard first-line TB treatment.

**Comorbidities** Presence of one or more additional following chronic conditions documented in the patient’s medical record at the time of TB diagnosis, in addition to HIV and TB: diabetes mellitus (DM), hypertension (HTN), asthma, chronic liver disease, chronic kidney disease, malignancy (cancer), chronic obstructive pulmonary disease (COPD), heart failure, epilepsy or other chronic condition not listed above but documented in the medical record.

#### Treatment Adherence

Adherence to antiretroviral therapy was operationally categorized into three distinct levels.

Good Adherence: Patient took ≥ 95% of the prescribed doses as scheduled.

Fair Adherence: Patient took between 85% and 94% of the prescribed doses.

Poor Adherence: Patient took < 85% of the prescribed doses, reflecting frequent missed appointments or significant gaps in treatment.

### Censoring criteria

Patients were censored if they met any of the following conditions: (1) were alive at the end of the 168-day follow-up period (administrative censoring); (2) were lost to follow-up (treatment interruption for ≥ 2 consecutive months) (3) transferred to another health facility for continuation of TB treatment. In all censoring cases, the last known date of follow-up (date of last clinic visit, date of transfer, or date of treatment completion was used as the censoring date.

### Handling of lost to follow-up and Transfer-outs

When participants were initially recorded as lost to follow-up (LTFU) but were subsequently traced or had later clinic visits documented, we updated the outcome information via traced or clinic-recorded dates. If the participant was identified as deceased, the date of death was used as the event date (and counted as an event if it occurred within 168 days (approximately six months of treatment initiation). If the participant was found to be alive, the participant was censored at the date of tracing or the last clinic visit, whichever occurred later, and was subjected to 168 days of administrative censoring, which was approximately six months.

For transferred-out participants, we also updated outcome information, and information from the receiving TB centre (as it is a routine procedure for reporting outcomes in the TB programme [19]) and documented in patient records and those living were censored at the last visit, and deaths within six months were recorded as events.

### Data quality control

In cases where discrepancies occurred between the HMIS and program tools with respect to key study variables, such as TB treatment initiation dates and patient outcomes, the TB registers were used as the primary tools; data collectors verified these onsite with input from the TB/HIV nurse. Trained data collectors worked closely with TB/HIV service nurses to ensure data accuracy and completeness during data collection; a National TB program staff member supervised the completion of the data extraction process, working with researchers and the University of Rwanda Research and Innovation Department to ensure consistency with standard operating procedures. In addition, the e-TB system-generated identifier helps prevent duplication of data during extraction.

### Statistical analysis

Baseline characteristics were summarized via descriptive statistics. Categorical variables are expressed as frequencies with percentages, whereas continuous variables are expressed as medians and (IQRs). The survival probabilities over 168 days were estimated via the Kaplan–Meier method, with group differences compared via the log-rank test. Survival time was calculated from TB treatment initiation to death or censoring.

Stepwise variable selection was used to identify predictors of mortality. Baseline categorical variables were screened for crude associations with mortality via the chi-square test. Variables of interest that demonstrated a significance level of *p* < 0.25 were considered potential candidates. These candidate variables were then analysed individually via bivariate Cox proportional hazards regression models to estimate crude hazard ratios (HRs) and associated 95% confidence intervals (CIs).

Variables of interest meeting the *p* < 0.25 criterion in bivariate analysis were then included in the multivariable Cox regression model to determine significant and nonsignificant predictors of mortality. In the final model, variable significance was determined as *p* < 0.05.

**Fig. 1:**
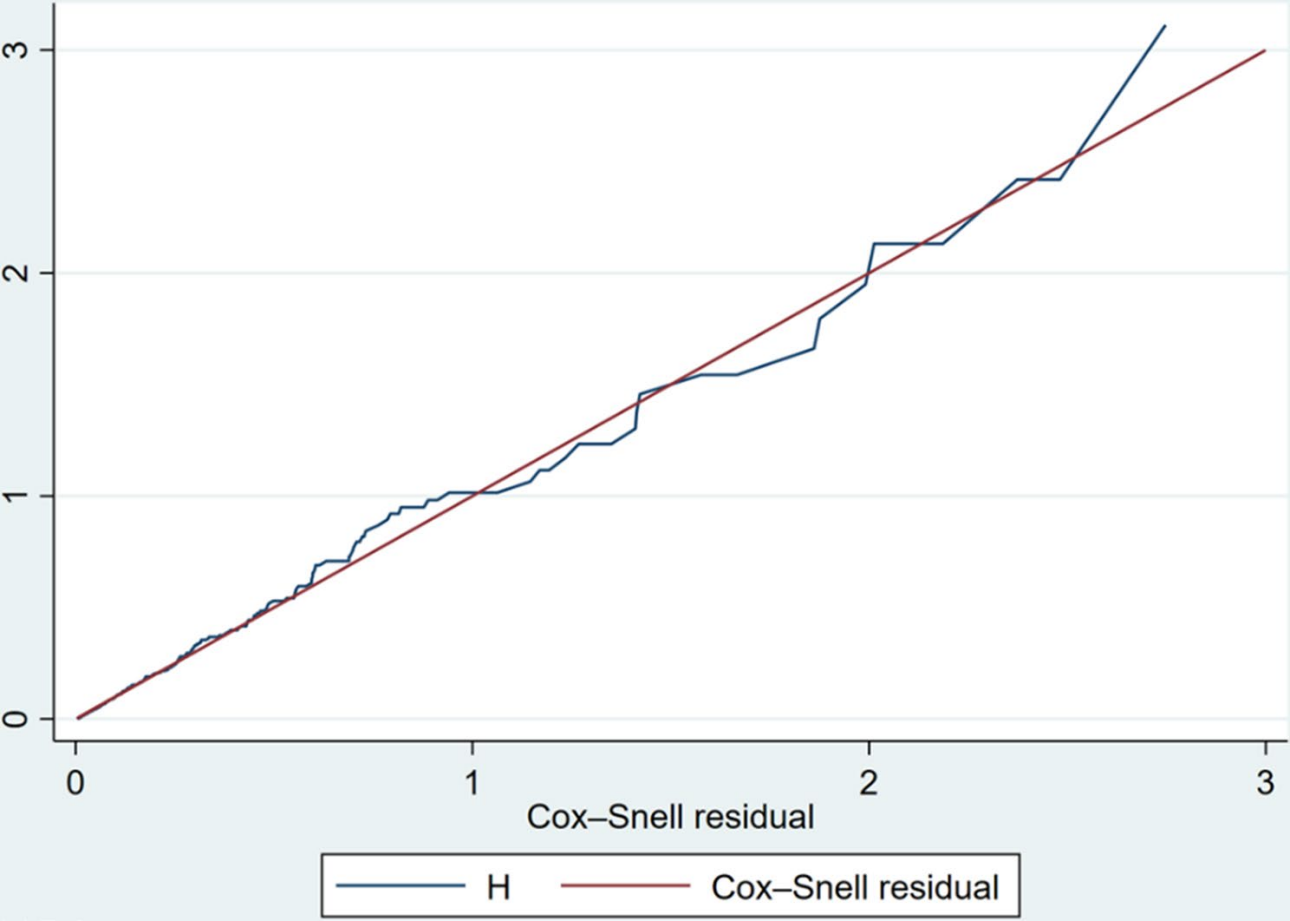
Cox–Snell residual plot for the proportional hazards model assessing TB/HIV coinfection mortality

The assumption of proportional hazards was evaluated via Schoenfeld residuals, with a nonsignificant global test (χ² = 22.79, df = 21, *p* = 0.3554), confirming that the assumption was satisfied. To further investigate the adequacy of the model, we used the Cox–Snell residuals, and the cumulative hazard function fell closely along the 45° reference line, indicating a good fit to the data (Fig. 1). Multicollinearity was checked via the variance inflation factor (VIF), with all covariates well below 5 (mean VIF = 1.16), signifying no problematic multicollinearity.

All analyses were conducted via Stata version 17 (Stata Corp, College Station, TX, USA).

### Ethical consideration

The study protocol was approved by the Institutional Review Board (IRB) and the University of Rwanda Ethics Committee before beginning the study (CMHS/IRB/185/2025).

The health facilities in Kigali provided appropriate approval to access their data, and all available patient records were anonymized to guarantee the privacy of the patients whose records were used for this study. Data access was limited to the research staff conducting this study.

As a retrospective cohort study that examines the use of secondary data extracted from the routine medical records of patients, it was not possible to obtain informed consent from each patient individually. As a result, the ethics committee approved a waiver for the need for informed consent because there would be no direct contact or intervention with any patient and did not include the collection of new personally identifiable information.

## Results

**Fig. 2:**
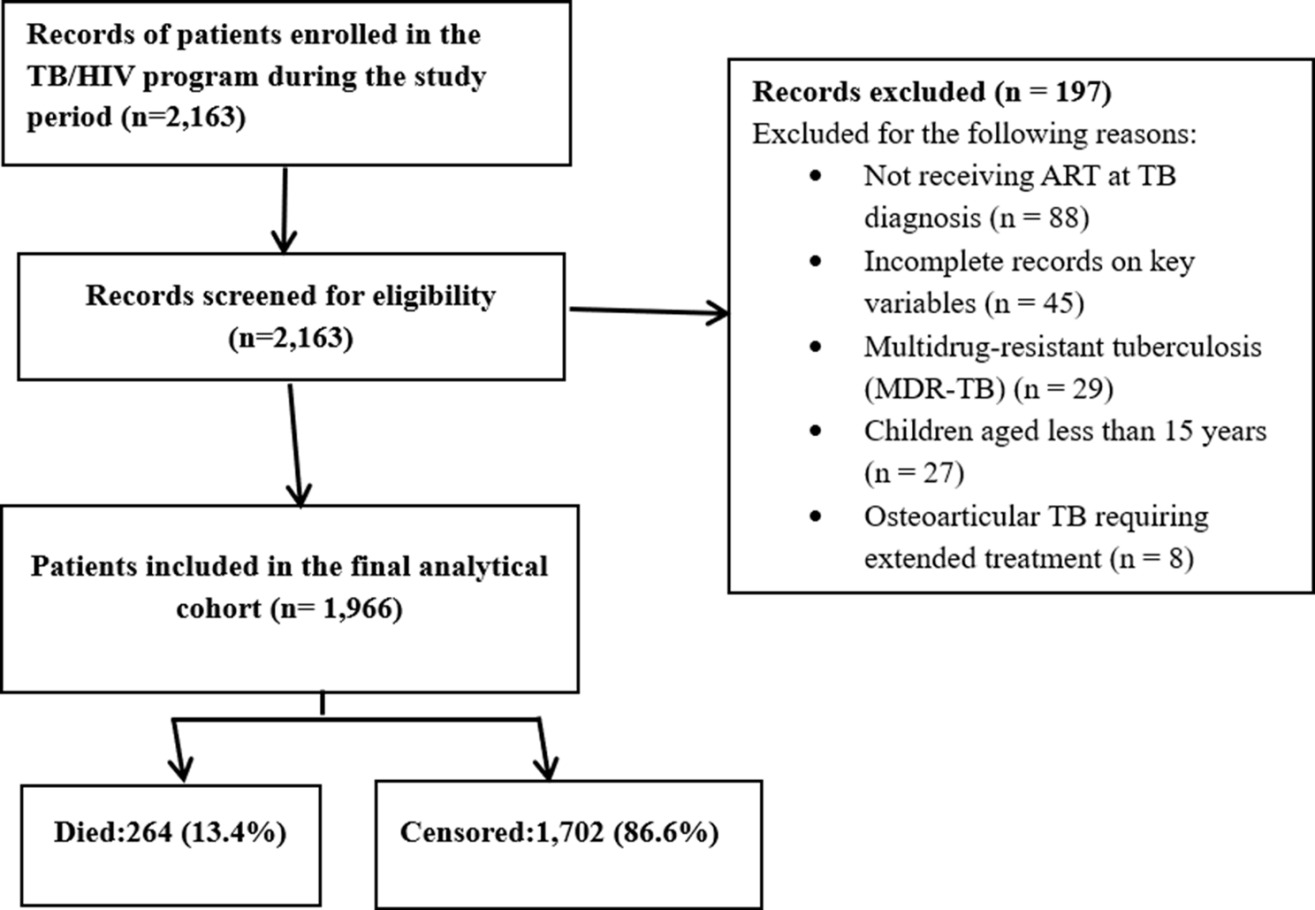
Consort flow chart of patient selection for the study

A total of 2,163 patients were enrolled in the TB/HIV program during the study period. Of these 197 patients were excluded from the final analysis for the following reasons: 88 were not receiving antiretroviral therapy (ART) at the time of TB diagnosis, 45 had incomplete information on key variables (including TB diagnosis date, treatment initiation, or outcomes), 29 had multidrug-resistant tuberculosis (MDR-TB), 27 were children aged less than 15 years, and 8 had osteoarticular TB requiring extended treatment duration beyond the standard protocol (Fig. 2). Hence, the study included 1,966 patients with TB/HIV co-infection, of whom 264, 13.4% (95% CI: 11.9–15.0), died during the follow-up period, while 1,702 patients (86.6%) were censored. The median age at baseline was 39 years (IQR: 33–46).

Most of the participants were male (1298, 66.0%) and were in the 35–44 years age group (738, 37.5%), followed by those aged ≥ 45 years (607, 30.9%). Two-thirds were either married or cohabiting (1305; 66.3%), whereas 28.7% were single. In terms of educational status, the majority had primary education (1087, 55.3%), 663 (33.7%) had secondary education, 159 (8.1%) were illiterate, and only 57 (2.9%) had a college or university education. More than half of the 1086 (55.2%) were in the informal sector, while 808 (41.1%) were unemployed and only 72 (3.7%) were formally employed.

Most patients had bacteriologically confirmed TB (1,579 (80.3%)), and pulmonary TB was the most common disease site (1,646 (83.7%)). Cotrimoxazole prophylaxis was started in 1249 (63.5%) patients, and nearly half of the 925 (47.0%) patients had received tuberculosis preventive therapy (TPT). The majority of 1832 (93.2%) reported no comorbidities.

With respect to HIV care, 1732 (88.1%) of the participants had good ART adherence, while 163 (8.3%) had poor adherence, and 3.6% had fair adherence. At baseline, 14.2% of patients had a CD4 count < 200 cells/mm³, and 27.1% were underweight (BMI < 18.5 kg/m²). (Table 1)

**Table 1:** Baseline characteristics of patients with TB/HIV co-infection and their survival status (2019–2024, Kigali city, Rwanda)

Characteristic	Total *n* (%)	Censored *n* (%)	Dead *n* (%)	*p* value
**Sex**				
Female	668 (34.0)	572 (85.6)	96 (14.4)	
Male	1298 (66.0)	1130 (87.1)	168 (12.9)	0.379
**Age group**				
15–24 years	109 (5.5)	94 (86.2)	15 (13.8)	
25–34 years	512 (26.0)	452 (88.3)	60 (11.7)	
35–44 years	738 (37.5)	677 (91.7)	61 (8.3)	
≥ 45 years	607 (30.9)	479 (78.9)	128 (21.1)	< 0.001
**Marital status**				
Married/cohabiting	1305 (66.3)	1152 (88.3)	153 (11.7)	
Single	564 (28.7)	473 (83.9)	91 (16.1)	
Widowed	70 (3.6)	54 (77.1)	16 (22.9)	
Divorced	27 (1.4)	23 (85.2)	4 (14.8)	0.007
**Education**				
University/college	57 (2.9)	51 (89.5)	6 (10.5)	
Secondary	663 (33.7)	617 (93.1)	46 (6.9)	
Primary	1087 (55.3)	905 (83.3)	182 (16.7)	
Illiterate	159 (8.1)	129 (81.1)	30 (18.9)	< 0.001
**Occupation**				
Formal employment	72 (3.7)	63 (87.5)	9 (12.5)	
Informal sector	1086 (55.2)	944 (86.9)	142 (13.1)	
Unemployed	808 (41.1)	695 (86.0)	113 (14.0)	0.825
**TB confirmation method**				
Clinical diagnosis	387 (19.7)	290 (74.9)	97 (25.1)	
Bacteriological	1579 (80.3)	1412 (89.4)	167 (10.6)	< 0.001
**Site of disease**				
Extrapulmonary	320 (16.3)	235 (73.4)	85 (26.6)	
Pulmonary	1646 (83.7)	1467 (89.1)	179 (10.9)	< 0.001
**Cotrimoxazole use**				
Yes	1249 (63.5)	1103 (88.3)	146 (11.7)	
No	717 (36.5)	599 (83.5)	118 (16.5)	0.003
**Comorbidities**				
No	1832 (93.2)	1643 (89.7)	189 (10.3)	
Yes	134 (6.8)	59 (44.0)	75 (56.0)	< 0.001
**TPT uptake**				
Yes	925 (47.0)	853 (92.2)	72 (7.8)	
No	1041 (53.0)	849 (81.6)	192 (18.4)	< 0.001
**ART adherence**				
Good	1732 (88.1)	1540 (88.9)	192 (11.1)	
Poor	163 (8.3)	120 (73.6)	43 (26.4)	
Fair	71 (3.6)	42 (59.2)	29 (40.8)	< 0.001
**BMI**				
Normal (> 18.5)	1433 (72.9)	1307 (91.2)	126 (8.8)	
Underweight (< 18.5)	533 (27.1)	395 (74.1)	138 (25.9)	< 0.001
**CD4 count**				
< 200 cells/mm³	280 (14.2)	210 (75.0)	70 (25.0)	
≥ 200 cells/mm³	1686 (85.8)	1492 (88.5)	194 (11.5)	< 0.001

### Survival analysis

During a total follow-up of 295,838 person-days, an overall incidence of 0.89 deaths per 1,000 person-days was observed. The median survival time was 168 days, and most patients survived the follow-up period.

**Fig. 3:**
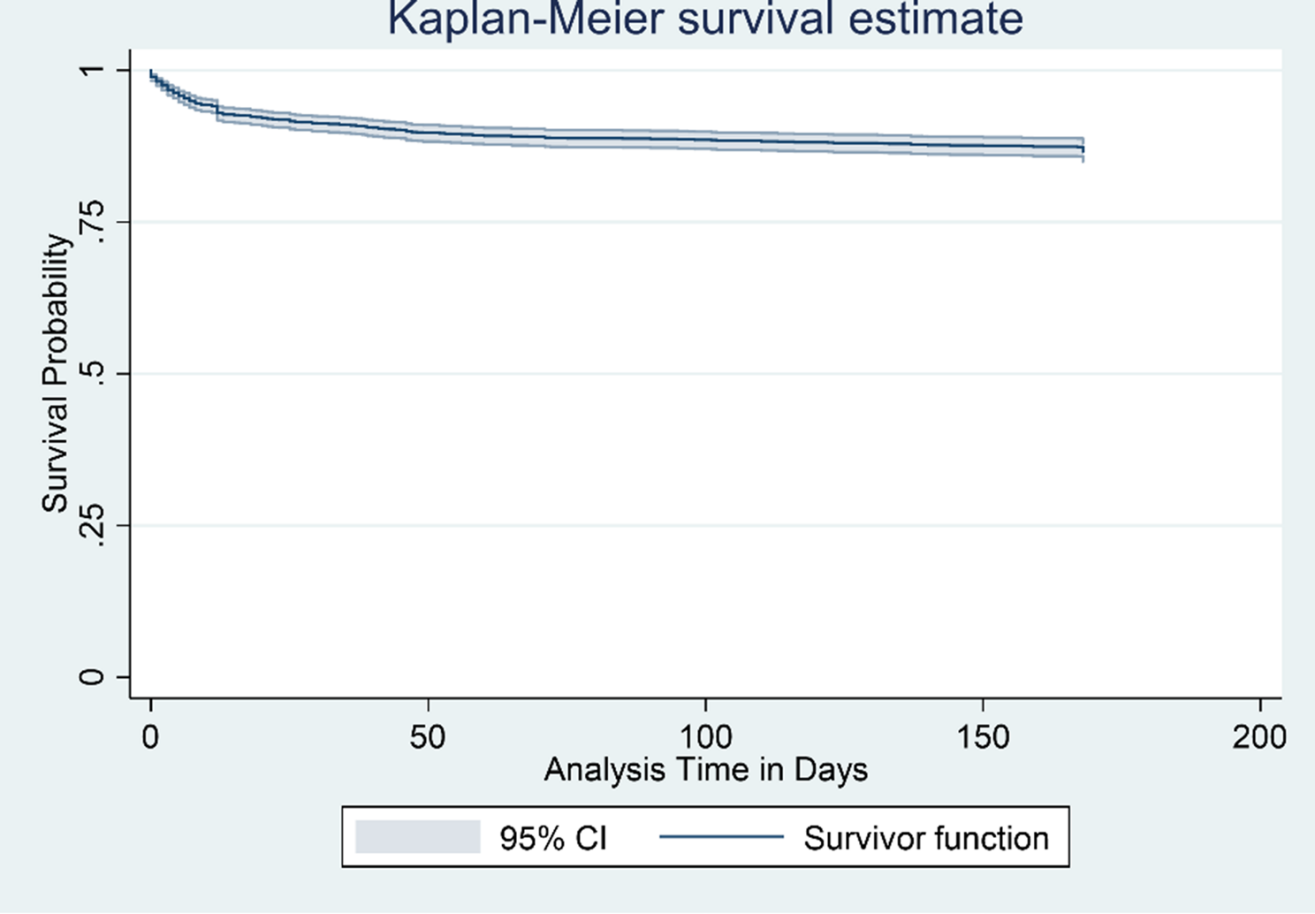
Overall Kaplan‒Meier survival estimates for the cohort of patients with TB/HIV co-infection over the study period

The Kaplan–Meier curve revealed a rapid decline in survival within the first 56 days of treatment, indicating that most of the deaths (78.78%) occurred early in the treatment period (Fig. 3). The probability of survival decreased from 100% at treatment initiation to 91.5% at 28 days (95% CI: 90.1–92.6) and 86.6% at 168 days (95% CI: 84.9–88.0). (Table 2).

**Table 2:** Kaplan–meier survival probability and incidence of mortality among patients with TB/HIV co-infection in Kigali, 2019–2024

Time (days)	Number at Risk	Failures	Survival Probability (%)	95% CI	Incidence Rate (per 1,000 person-days)
28	1,799	168	91.5	90.1–92.6	3.34
56	1,760	40	89.4	87.9–90.7	0.81
84	1,747	12	88.1	87.3–90.1	0.25
112	1,738	10	88.3	86.8–89.6	0.21
140	1,725	12	87	86.2–89.1	0.25
168	1,718	22	86.6	84.9–88.0	0.46

### Survival analysis by key clinical factors

**Fig. 4:**
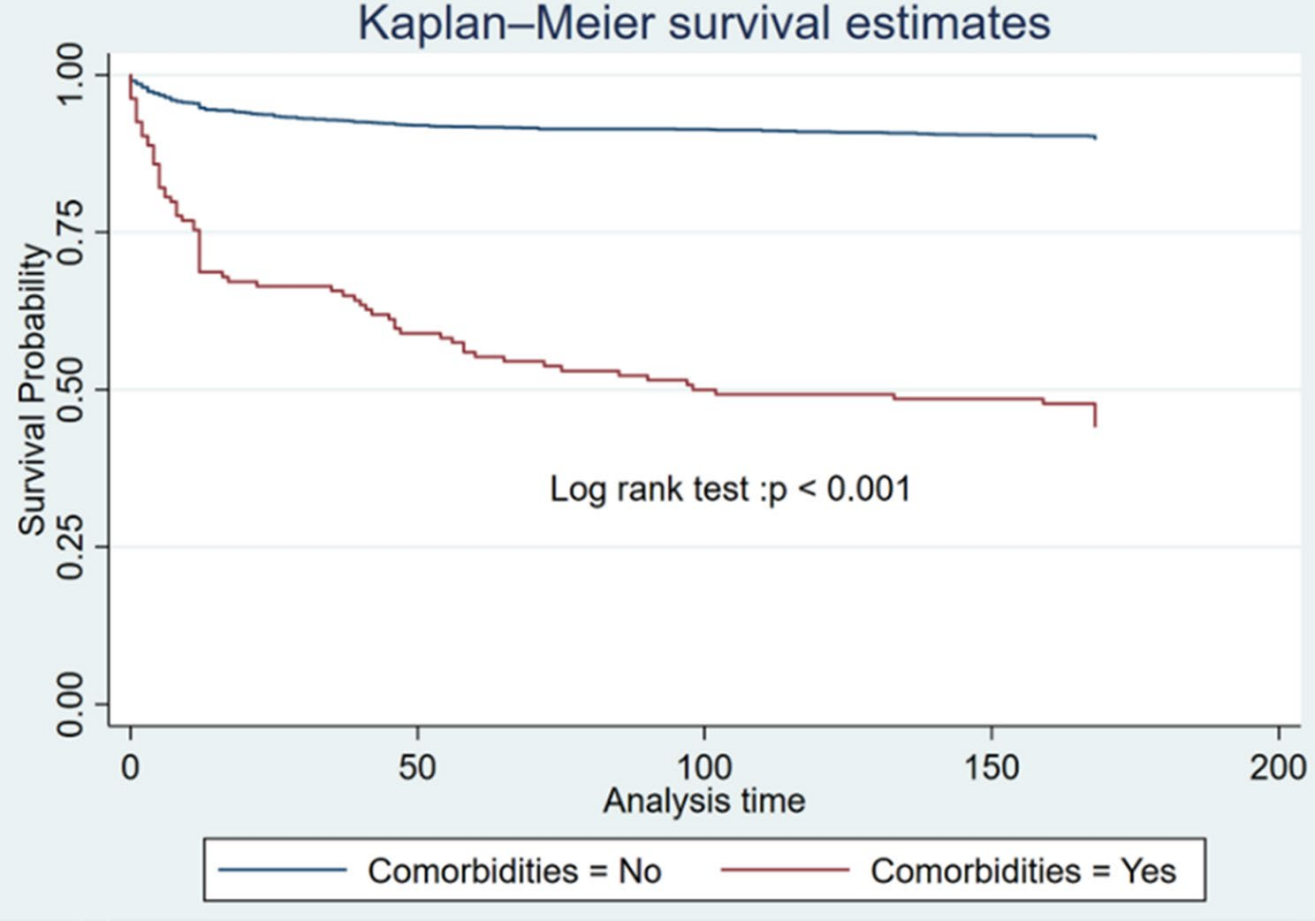
Kaplan–Meier survival estimates, stratified by comorbidity status among patients with TB/HIV co-infection in Kigali, July 2019–March 2024

The results of the Kaplan‒Meier survival analysis indicated a much lower survival probability for patients with comorbidities than for those without comorbidities (log-rank test: *p* < 0.001). For example, at the time of analysis, for patients who had no comorbidities, the estimated survival probability was approximately 93%, whereas for patients who had comorbidities, the estimated survival probability decreased to approximately 50%. This large gap in survival probability indicates the detrimental prognostic effect of preexisting health conditions on patient survival. (Fig. 4)

**Fig. 5:**
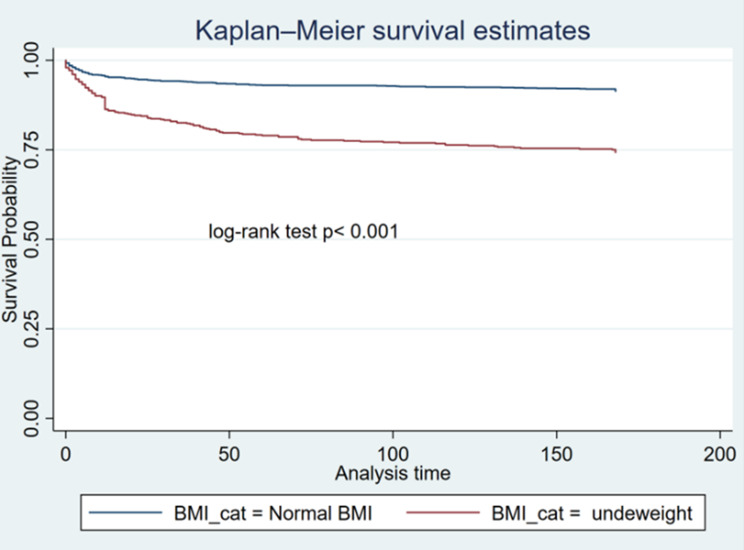
Kaplan‒Meier survival curves stratified by baseline nutritional status among patients with TB/HIV co-infection

Similarly, the Kaplan‒Meier survival estimation indicated a significantly lower survival probability for patients who were underweight than for patients with a normal BMI (log-rank test: *p* < 0.001). The estimated survival probability for the underweight category had plateaued at approximately 75% risk of dying, whereas the normal BMI category had an estimated risk of dying of approximately 5%. These estimates indicate that poor nutritional status is a negative prognostic factor. (Fig. 5)

**Fig. 6:**
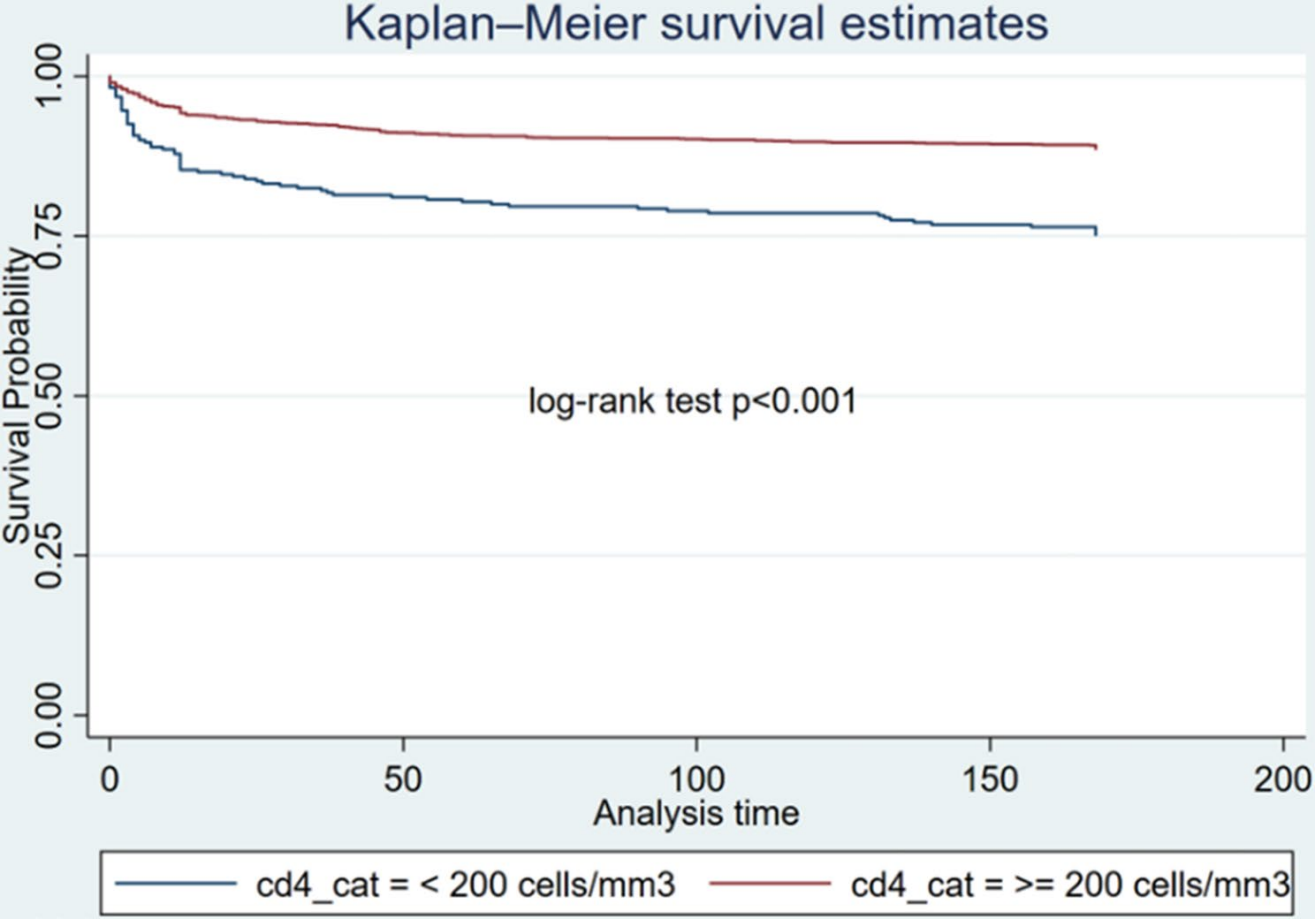
Kaplan‒Meier survival curves stratified by baseline CD4 + T-cell count among patients with TB/HIV co-infection

Survival analysis stratified by the baseline CD4 count revealed a highly statistically significant difference in survival probability between the groups (log-rank test: *p* < 0.001; Fig. 6). Patients with a CD4 count of < 200 cells/mm³ experienced poorer survival, with an estimated probability of survival of approximately 75% at the end of the analysis time, versus nearly 90% for patients with a CD4 count of ≥ 200 cells/mm³, confirming that being severely immunocompromised is a major determinant of outcome (Fig. 6).

### Risk factors for mortality among patients with TB/HIV co-infection in Kigali, 2019–2024

In the multivariable Cox regression model, several independent predictors of mortality among patients with TB/HIV co-infection were identified. Coexisting comorbidities were the strongest predictor, with a more than threefold increase in the hazard of death (HR = 3.18; 95% CI: 2.27–4.45, *p* < 0.001). Underweight status, defined as a body mass index (BMI) < 18.5, was also categorized as a strong predictor of mortality, with patients classified as underweight having a 2.72-fold increased hazard of death compared with patients classified with a normal BMI (95% CI: 2.12–3.50, *p* < 0.001). A CD4 count < 200 cells/mm³ was also associated with an increased 2.30 hazard of death (95% CI: 1.74–3.05, *p* < 0.001). (Table 3)

**Table 3:** Risk factors for mortality among patients with TB/HIV co-infection in Kigali, 2019–2024

Characteristic	Crude HR (95% CI)	*P* value	Adjusted HR (95% CI)	Adjusted *P* value
**Age Group**				
15–24 years	1		1	
25–34 years	0.86 (0.49–1.51)	0.59	1.02 (0.57–1.80)	0.958
35–44 years	0.59 (0.34–1.04)	0.068	1.16 (0.61–2.19)	0.656
45 years and above	1.61 (0.94–2.75)	0.08	2.21 (1.18–4.11)	**0.013**
**Marital Status**				
Married/Co habitation	1		1	
Single	1.41 (1.09–1.82)	0.01	1.96 (1.37–2.80)	**< 0.001**
Widowed	2.05 (1.22–3.43)	0.006	0.87 (0.51–1.49)	0.616
Divorced	1.32 (0.49–3.55)	0.588	1.53 (0.56–4.16)	0.408
**Method of TB Confirmation**				
Bacteriologically confirmed	1		1	
Clinically diagnosed	2.55 (1.99–3.28)	< 0.001	1.25 (0.90–1.74)	0.18
**Site of Disease**				
Pulmonary	1		1	
Extra pulmonary	2.67 (2.06–3.46)	< 0.001	1.62 (1.15–2.28)	0.006
**On Cotrimoxazole**				
Yes	1		1	
No	1.46 (1.14–1.86)	0.002	1.65 (1.29–2.13)	< 0.001
**Comorbidities**				
No	1		1	
Yes	7.13 (5.45–9.33)	< 0.001	3.18 (2.27–4.45)	< 0.001
**TPT Uptake**				
Yes	1		1	
No	2.51 (1.92–3.29)	< 0.001	1.87 (1.38–2.54)	< 0.001
**Adherence to HIV Treatment**				
Good	1		1	
Poor	2.55 (1.83–3.55)	< 0.001	1.34 (0.93–1.93)	0.116
Fair	4.30 (2.91–6.35)	< 0.001	1.89 (1.25–2.87)	0.003
**BMI Category**				
Normal BMI	1		1	
Underweight	3.22 (2.53–4.10)	< 0.001	2.72 (2.12–3.50)	< 0.001
**CD4 Count Category**				
≥ 200 cells/mm³	1		1	
< 200 cells/mm³	2.36 (1.79–3.10)	< 0.001	2.30 (1.74–3.05)	< 0.001

## Discussion

This retrospective cohort study analysed the survival and risk factors for mortality among patients with TB/HIV coinfection in Kigali city, Rwanda, over a recent five-year period. We found a mortality rate of 13.4% (95% CI: 11.9–15.0) over approximately 295,838 persons‒days of follow-up, corresponding to an overall survival probability of 86.6% (95% CI: 84.9–88.0). These findings suggest that many patients with TB/HIV co-infection successfully complete treatment. This survival probability suggests that while treatment services are generally effective, gaps in early diagnosis, the initiation of treatment with severe immunosuppression, poor management of comorbidities, or a lack of adherence support, among other potential gaps, remain.

The mortality rate observed in our cohort was relatively low compared with that reported in other areas. For example, in a published meta-analysis of patients in Ethiopia who were coinfected with TB and HIV, the average mortality rate for this patient population was approximately 16.67% [8]. Our findings suggest that the comparatively lower mortality rate in our cohort from the city of Kigali may be explained by the effectiveness of integrated TB and HIV services in Rwanda and large-scale ART coverage combined with continuing programmatic support through the “one-stop” approach for service delivery [20, 21]. In addition, Rwanda’s continuing efforts to invest in integrated care and continue the development of preventive therapies for people living with HIV may support these positive outcomes. However, the fact that the remaining approximately 13–14% do not survive illustrates continued vulnerabilities in this population.

The majority of deaths in our cohort (78.8%) occurred within the first 56 days of TB treatment, indicating very early mortality among patients with TB/HIV coinfection. This finding is consistent with previous studies from sub-Saharan Africa, which reported the same trend of high mortality shortly after the initiation of treatment. For example, a retrospective cohort study in Ethiopia revealed that mortality among patients with TB/HIV coinfection occurred in the early phase following treatment initiation [15]. Similarly, a retrospective study conducted in Cameroon reported a significant decrease in survival rates during the first month of TB treatment and that the majority of deaths occurred within the first month of treatment initiation [22]. During the Ebola outbreak in Guinea, 52% of patients with TB/HIV coinfection who died were documented as having died within eight weeks following the commencement of TB treatment [23].

Several clinical and programmatic factors may explain this high early mortality. Many patients initiate TB treatment at an advanced stage of both infections, characterized by a high mycobacterial burden, profound immunosuppression often reflected by very low CD4 + T-cell counts and a high prevalence of severe comorbidities such as anaemia and malnutrition. In addition, the early phase of TB therapy may be complicated by immune reconstitution inflammatory syndrome (IRIS), a dysregulated inflammatory response following ART initiation that can lead to clinical deterioration and death. Importantly, early mortality also reflects substantial diagnostic delays prior to treatment initiation. Many patients experience prolonged periods of illness before diagnosis and present for care with advanced or disseminated disease, including extrapulmonary TB, and irreversible physiological compromise. This “late presentation” phenomenon implies that even prompt initiation of effective TB treatment and ART may be insufficient to reverse advanced disease processes. Therefore, the intensive phase of TB therapy provides information regarding the severity of the disease. The high number of deaths that occur during this phase of treatment underscores the need for improved case detection, rapid treatment initiation, and clinically intensified monitoring during the first few weeks of TB therapy.

In this cohort, comorbidities were the most significant predictor of mortality. More than two-thirds of patients with comorbid conditions have chronic diseases. Hypertension was present in approximately 16% of patients, with diabetes mellitus in 10% of patients and asthma in a smaller proportion of patients. More than 66% of the individuals who had comorbid conditions had very severe conditions, such as liver disease, kidney disease, and cancer. Patients with these comorbid conditions have a threefold increase in mortality risk. This finding supports previous evidence from sub-Saharan Africa showing that people with TB/HIV coinfections who have multiple chronic illnesses have poorer outcomes than those who do not [24]. Chronic illnesses may accelerate the progression of TB through chronic inflammation, disruption of normal immune responses, drug interactions, and increased complexity in the administration of treatments. In urban areas such as Kigali, where there is an increasing prevalence of noncommunicable diseases, these results reinforce the need to integrate and coordinate tuberculosis (TB), human immunodeficiency virus (HIV), and chronic disease care to reduce preventable deaths.

Another prominent predictor of mortality among patients with TB/HIV coinfection was undernutrition, with underweight patients experiencing significantly greater hazards of mortality. Evidence suggests that malnutrition can alter the pharmacokinetics of drugs used to treat TB as a result of decreased intestinal absorption, which changes the way the drug is metabolized and distributed in the body and could result in lower-than-expected drug levels or an increased risk of toxic effects [25]. The combined impact of these factors could help explain the high mortality rates among undernourished patients with TB/HIV coinfection. Therefore, the results of this study highlight the need for regular nutritional assessments, timely nutritional interventions, and close monitoring of treatment response and toxicity as part of a comprehensive TB/HIV treatment program.

Advanced immunosuppression was strongly associated with increased mortality in this cohort, as shown by a CD4 count less than 200 cells/mm³. This finding is consistent with other reports that support that an individual’s initial CD4 count is strongly associated with the individual’s risk of dying from TB/HIV coinfection [15, 26]. The initial CD4 count reflects a high level of immunosuppression and thus leaves individuals vulnerable to opportunistic infections, leading to severe disease, disseminated tuberculosis (TB), and treatment complications. Studies in sub-Saharan Africa indicate a higher level of mortality for patients who present for care with advanced immunosuppression, and many still face barriers such as delayed presentation to HIV services and delayed immune recovery as persistent challenges despite expanded testing and treatment programs [27]. Owing to the continued vulnerability to severe disease and secondary infections, it is extremely important that any effort to improve survival for patients who have both TB and HIV includes increasing the timing of HIV diagnosis and linkage to care.

Age 45 and older was associated with an increased risk of mortality, as has previously been established in prior research, which has shown poorer health outcomes in adults over the age of 45 infected with TB/HIV because many factors contribute to the death of patients with TB/HIV co-infection, including the effects of immune senescence on aging, the accumulation of additional illnesses (comorbidities), and a decreased ability of the body to recover from illness (physiological reserve) [8]; therefore, the biological and clinical challenges experienced by older adults necessitate an age-sensitive approach in providing TB/HIV management, which includes close monitoring and individualized patient support to promote treatment adherence and ultimately improve survival.

Patients who did not receive tuberculosis preventive therapy (TPT) had a significantly greater risk of death than did those who did. Moreover, several studies have demonstrated that the prevalence of mortality is significantly greater among people living with advanced HIV disease who have not utilized TPT than among those who have utilized TPT [28, 29]. In Rwanda, large-scale implementation of TPT among people living with HIV intensified in approximately 2021, following revisions to the TPT guidelines, which recommended routine TPT provisions for all eligible PLHIV after the exclusion of active TB [30, 31]. Prior to this scale-up, many PLHIV enrolled in care were not systematically initiated on TPT, increasing their vulnerability to developing active TB and its associated complications. This programmatic context helps explain why a lack of TPT uptake in our cohort was associated with increased exposure to TB and increased mortality. Strengthening and sustaining early TPT initiation, particularly at the time of ART initiation and in accordance with national guidelines, remains critical for reducing TB-associated mortality among coinfected individuals.

Patients who did not receive cotrimoxazole prophylaxis and those with fair ART adherence had a significantly greater risk of mortality. A lack of cotrimoxazole preventive therapy has been identified as an independent predictor of death among patients with TB/HIV coinfection in African settings [8, 16]. The national HIV treatment guidelines state that all patients with TB/HIV coinfection in, regardless of their CD4 count, should receive routine cotrimoxazole prophylaxis, as it protects against bacterial infections and the development of opportunistic infections or severe bacterial illness [20]. Consequently, the higher mortality observed in patients not receiving cotrimoxazole indicates a failure to fully implement this fundamental element of the HIV care package. Improvements in survival for these patients can be achieved through improvements incontinuous access to cotrimoxazole prophylactic treatment.

Patients who have fair ART adherence have been shown to have a significantly greater risk of mortality compared to those with good adherence. This finding highlight that any deviations from optimal adherence may compromise the effectiveness of treatment resulting in reduced viral suppression and incomplete recovery of the immune system. In addition, a patient’s inability to adhere adequately to their ART regimen would have limited their ability to achieve viral suppression and immune recovery, putting them at increased risk for poor outcomes. This underscores the importance of maintaining consistently high levels of adherence and improvements in adherence counselling.

The presence of extrapulmonary TB (EPTB) is associated with increased mortality, reflecting more advanced or disseminated disease, particularly among immunocompromised patients. Research from high-burden settings confirms that extrapulmonary TB is associated with greater mortality than pulmonary TB is, a risk that is amplified by HIV coinfection, advanced immunosuppression (low CD4), age greater than 45 years, and malnutrition [8, 32]. Owing to the low number of bacilli present in EPTB patients and their nonspecific clinical presentation, delays in diagnosis are common; as a result, treatment is often initiated later, and outcomes are worse [32]. Therefore, it is critically important to increase the use of rapid and sensitive diagnostic tests for the early identification of EPTB in individuals living with HIV, particularly those with the most severe levels of immunosuppression, to reduce mortality from EPTB.

### Cues to action

The findings from the study suggest a number of different actions that can be taken to improve survival of patients with TB/HIV coinfection in urban locations with a high disease burden like Kigali. The first action is to strengthen early tuberculosis case detection through routine TB screening among people living with HIV and timely HIV diagnosis to reduce delays in treatment initiation and prevent the advanced presentation of TB. Intensified monitoring of patients undergoing TB treatment during the first two months of TB treatment is crucial, as most of deaths occurred during this period. This may consist of closer follow-up visits, rapid management of complications, early detection of severe comorbidities, and a nutritional assessment.

The integrated management of comorbidity conditions such as diabetes mellitus, hypertension, chronic kidney disease, and liver disease should be reinforced within TB/HIV care programs to reduce the risk of mortality. Integrated nutritional support, including routine nutritional screening and supplementation for undernourished patients, should be emphasized.

In addition, there is a need to improve adherence to ART and ensure continuous access to CPT and TPT among people living with HIV. Community-based patient tracing systems, adherence counselling, and differentiated service delivery models should all be used to improve retention in TB/HIV management services. Finally, increasing training and access to rapid and sensitive TB diagnostic tools for extrapulmonary TB and advanced HIV disease could facilitate earlier diagnosis and treatment among severely immunocompromised patients. Collectively, these interventions should be implemented to reduce the early mortality among Patients with TB/HIV coinfection in Kigali city, Rwanda and similar settings.

### Strengths and limitations

A key strength of this study is its large urban cohort and long follow-up period. By utilizing a relatively large citywide population with participants recruited from all tuberculosis treatment centres in Kigali, the results are highly valid and generalizable to similar urban populations of patients with TB/HIV co-infection in Rwanda. Additionally, the survival analysis allowed the determination of independent predictors of mortality over time.

On the other hand, several limitations must be considered. First, the retrospective design uses routinely collected programmatic data; therefore, there may have been incomplete documentation, misclassification, or errors in recording. Second, there are clinically important variables that were not consistently recorded in the routine records used in this analysis. These include viral load measurements, opportunistic infections due to HIV, various biological test results, and detailed socioeconomic indicators that may have affected or changed the association with mortality, as reported in this study.

Furthermore, a significant limitation is the absence of a comparison group (e.g., TB patients without HIV infection). The lack of such a group limits our ability to contextualize the absolute magnitude of the observed risks or to compare survival outcomes against other relevant patient populations. Consequently, the identified predictors should be interpreted as factors specifically influencing mortality within the high-risk co-infected population, rather than relative to the general TB patient population.

Additionally, the study’s limitations include the fact that it was performed in an urban area (Kigali), which may limit the generalizability of the results to rural locations in Rwanda or other locations with different access to healthcare or demographic characteristics and disease patterns. Finally, the diagnosis of extrapulmonary TB often relies on clinical criteria in resource-limited settings, which may result in misclassification or under detection, particularly among severely immunosuppressed patients. Future studies involving longitudinal data collection, prospective designs with appropriate comparison cohorts, routine monitoring of viral load and longer follow-up periods will help improve upon the limitations encountered in this study and provide stronger evidence concerning mortality risk for patients with TB/HIV coinfection.

## Conclusion

The findings from this large retrospective cohort study revealed that although integrated TB/HIV services are available throughout the city of Kigali, a high percentage (13.4%) of patients have died from TB/HIV coinfection. Most deaths occurred within the first 2 months of starting treatment, indicating a “critical” period early in care, during which patients are highly vulnerable to death.

Risk factors for mortality included the presence of comorbidities, underweight status, advanced immunosuppression (< 200 cd/mm³), extrapulmonary TB, not being on cotrimoxazole prophylaxis, lack of tuberculous preventive therapy, poor adherence to ARV treatment, and age.

These findings suggest that patient survival outcomes are influenced by access to TB and HIV treatment, by the severity of their disease when they present for care, and by the level of prevention and supportive care they receive.

The key to decreasing preventable deaths is to strengthen the early detection of patients with TB/HIV coinfection. Timely initiation of ART, nutritional support, good management of comorbidities, support for adherence to ARVs, and the ability to follow national guidelines with respect to the use of cotrimoxazole prophylaxis are important. In addition, it is equally important to expand the early implementation of preventive therapy for tuberculosis when patients start ART. High-risk populations, especially older adults and those who present with advanced immunosuppression or extrapulmonary disease, should be prioritized. Implementing these strategies could reduce early mortality.

## Data Availability

The datasets generated and analysed during the current study are available from the corresponding author upon reasonable request.

## Abbreviations

ART: Antiretroviral therapy
BMI: Body mass index
TPT: Tuberculosis Preventive Treatment
LTFU: Lost to follow-up
HIV: Human immunodeficiency virus
HR: Hazard ratio
PLHIV: people living with HIV
HIV: human immunodeficiency virus
TB: tuberculosis
MTB: Mycobacterium tuberculosis
RIF: Rifampicin
